# Short group psychoeducation followed by daily electronic self-monitoring in the long-term treatment of bipolar disorders: a multicenter, rater-blind, randomized controlled trial

**DOI:** 10.1186/s40345-019-0158-8

**Published:** 2019-11-04

**Authors:** Johannes Petzold, René Mayer-Pelinski, Maximilian Pilhatsch, Susan Luthe, Thomas Barth, Michael Bauer, Emanuel Severus

**Affiliations:** 1 Department of Psychiatry and Psychotherapy, Carl Gustav Carus University Hospital, Technische Universität Dresden, Fetscherstr. 74, 01307 Dresden, Germany; 2 Department of Psychiatry, Behavioral and Psychosomatic Medicine, Chemnitz Hospital, Flemmingstr. 2, 09116 Chemnitz, Germany

**Keywords:** Bipolar disorder, Psychotherapy, Self-management, Self efficacy, Self report, Recurrence, Quality of life

## Abstract

**Background:**

Despite various pharmacological and psychological treatment interventions, bipolar disorders rank among the leading causes of global disease burden. Group psychoeducation has been demonstrated an effective add-on to pharmacotherapy, but it may be difficult to implement in practice depending on the clinical setting and available human resources.

**Methods:**

Multicenter, rater-blind, randomized controlled trial to investigate the efficacy of a new intervention program consisting of an initial 6-week psychoeducation protocol plus a subsequent structured daily computer-based self-charting program (ChronoRecord) over 54 weeks in remitted patients with bipolar disorders. The control condition included non-structured group sessions followed by daily computer-based self-reports (unstructured like a diary). Both groups received treatment-as-usual.

**Results:**

Over 2 years, 41 mood episodes occurred in the experimental group (*n* = 39) compared to 27 in the control group (*n* = 34), without reaching statistical significance. Time to recurrence did not significantly differ between the experimental and control group (25% relapsed after 112 and 273 days, respectively). There were no significant group-by-time interactions in mood symptoms, quality of life, self-efficacy expectations or perceived involvement in care.

**Conclusions:**

Six weekly psychoeducational group sessions followed by daily self-monitoring via ChronoRecord for 54 weeks may not be superior to non-structured group meetings followed by unstructured self-reporting. Other psychotherapeutic interventions may be needed to optimize the treatment of patients with bipolar disorders, especially for those at later disease stages.

*Trial registration* Retrospectively registered at German Clinical Trials Register on May 24, 2019; DRKS00017319

## Background

Bipolar disorders (BD) are serious mental health conditions characterized by severe mood swings. Despite the availability of effective pharmacological treatments (National Collaborating Centre for Mental Health 2018), the long-term course of BD remains marked by frequent relapses and recurrences as well as subsyndromal symptoms (Treuer and Tohen 2010). As a consequence, BD are a leading cause of disability worldwide (Catala-Lopez et al. 2013), are associated with a high suicide rate (Nordentoft et al. 2011) and remain a major public health problem. In this context, psychoeducation (PE) manuals have been developed to empower patients to actively contribute to their treatment and thereby maintain remission and reach full recovery. This is to be achieved by comprehensively educating patients about BD with focusing on self-management skills. Typical topics covered are etiology, symptoms, course and treatment of BD. Improving coping strategies, adherence and early recognition of new episodes are important features. As an add-on to pharmacotherapy, group PE has been shown to reduce recurrences and prolong time to recurrences compared to an unstructured control condition (Colom et al. 2003a, b, 2009b). However, the program applied (Colom and Vieta 2006) has not reached dissemination in clinical practice (Hidalgo-Mazzei et al. 2018), possibly due to its length and associated costs. Since a large observational study using a within-individual analysis of registry data indicates the effectiveness of short-term group programs (Joas et al. 2019), these may help to expand the routine use of PE, yet there is insufficient evidence from randomized controlled trials (RCT).

We therefore investigated the efficacy of a 6-week PE program (Erfurth et al. 2005) followed by computer-based self-charting (Bauer et al. 2004) added to treatment-as-usual (TAU) in a multicenter RCT. We hypothesized that this combined intervention would result in less recurrences/relapses (primary outcome), longer time to recurrence as well as improved mood stability, quality of life, self-efficacy expectations and perceived involvement in care (secondary outcomes). The combination of a short course of PE with computer-based self-charting should have a synergistic impact. Computer-based self-charting may strengthen patients’ self-management abilities, thereby helping to maintain the knowledge and coping skills learned in PE for the long term. The combined intervention should promote a stronger role for patients in their treatment, result in a positive change in patients’ self-efficacy and enhance the patient-physician interaction. Improvements in coping skills may help to explain the reported effects of PE on time to recurrence and frequency of episodes that go beyond the enhancement of treatment adherence (Colom et al. 2003b; Miziou et al. 2015). Assessments in this study included individual coping skills as well as standard outcome measures of mood symptoms. The trial aimed to conduct the first RCT of PE in Germany and go beyond what has been demonstrated in the scientific literature by testing a combined non-pharmacological approach in the treatment of BD.

## Methods

### Study design

This is a multicenter RCT in a parallel 2-group design added to TAU with standard pharmacotherapy for patients with remitted BD. Using a computer-generated randomization, subjects were assigned to the experimental or control intervention. Only the group therapist was informed of the allocation. The duration of intervention per patient was to be 60 weeks (6 weeks PE or supportive counseling plus 54 weeks computer-based self-charting or diary, respectively). Each group was comprised of 5–10 persons, and group sessions lasted 90 min.

The experimental intervention included 6 weekly psychoeducational group sessions followed by daily self-monitoring via ChronoRecord (ChronoRecord Association, Fullerton, CA, USA) for 54 weeks. The PE program applied was developed for patients with BD covering the following modules: (1) definitions, (2) symptoms of the disease, (3) disease course, (4) treatment, (5) causes of the disease, (6) early signs of recurrence/relapse and staying healthy (Erfurth et al. 2005). ChronoRecord is a well-accepted and validated computer-based software used by patients with BD to report mood, sleep, life events, weight, menstrual data and psychiatric medication (Bauer et al. 2004, 2008). Patients were instructed to rate these items once a day (weight once a week) at the same time every day for the prior 24 h. To control for non-specific factors (i.e., contact with therapist, group activities, self-monitoring, length of program), the experimental intervention was compared to 6 weekly supportive non-structured group meetings in which no specific instructions were given. This supportive counseling was followed by daily unstructured computed-based self-reports for 54 weeks. Patients were instructed not to spend more than 5 min per day for these diary entries to match the time needed to do self-monitoring via ChronoRecord.

### Participants

Patients were recruited from hospitals in Berlin, Dresden and Chemnitz (all in Germany). The inclusion criteria were: (a) bipolar I or II disorder according to the Diagnostic and Statistical Manual of Mental Disorders (DSM) IV, (b) remission for at least 2 months (Young Mania Rating Scale [YMRS] < 6, Hamilton Depression Rating Scale [HDRS-17] < 8), (c) at least 1 recurrence/relapse according to DSM-IV in the past 3 years, (d) current pharmacotherapy of BD, (e) ≥ 18 years of age, (f) daily access to PC. Patients who did not have access to a PC were provided one throughout the study period. The exclusion criteria were: (a) current diagnosis of substance dependence, dementia or organic mental disorder, (b) diagnosis of antisocial personality disorder, (c) ultra-rapid cycling (≥ 12 episodes) in the past year, (d) currently receiving psychotherapy, (e) current participation in drug trial.

### Assessments

Trained research associates blind to group allocation conducted assessments at baseline (i.e., before study enrollment) and in 9 follow-up visits over 2 years (counting from first group session). Besides a clinical interview and the Structured Clinical Interview for DSM-IV (SCID; Wittchen et al. 1997), assessments of mood via YMRS (Young et al. 1978) and HDRS-17 (Hamilton 1960) were conducted at baseline, after the last group session and then every 3 months (counting from first group session). The last ratings were done 24 months after the first group session (i.e., 9 months after end of self-reporting). Quality of life, self-efficacy expectations and perceived involvement in care were assessed at baseline, after the last group session and then 6, 12, 15 and 24 months after the first group session. Outcome variables were assessed as follows: (a) recurrence/relapse frequency and time to first recurrence with YMRS, HDRS-17 (both referring to time of follow-up) and SCID-I (referring to time since last assessment), (b) mood symptoms with YMRS and HRDS-17, (c) quality of life with the 36-Item Short Form Survey (SF-36; Bullinger 1995), (d) self-efficacy expectations with the General Self-Efficacy Scale (GSE; Schwarzer 1994) and the Health Locus of Control Scale (HLOC; Ferring and Filipp 1989), (e) perceived involvement in care with a 14-item version of the Perceived Involvement in Care Scales (PICS; Lerman et al. 1990). Recurrence/relapse was defined as follows: (a) mania ≥ 20 on YMRS, (b) hypomania ≥ 12 on YMRS, (c) depression ≥ 17 on HDRS-17, d) mixed state ≥ 20 on YMRS+ ≥ 12 on HDRS-17, (e) any affective episode meeting DSM-IV criteria (assessed with SCID-I) that occurred between assessments. Patients were not excluded for recurrences/relapses but the follow-up continued. Stopping self-monitoring for more than 60 days (except time of inpatient treatment) or attending any psychological treatment during the trial led to exclusion.

### Statistics

We used SPSS Statistics Version 25 (IBM, Armonk, NY, USA) and R (R Foundation for Statistical Computing, Vienna, Austria) for all analyses assuming 2-tailed significance at *p* < 0.05. We compared the participant characteristics of the 2 groups using Pearson’s Chi-square test for categorical variables applying Fisher’s exact test if needed and the independent t-test for continuous variables applying the Mann–Whitney U test if needed. The efficacy of the experimental intervention was established in an intention-to-treat analysis with last observation carried forward for missing data applying random forests when needed. As we were primarily interested in the impact of PE, only recurrences that occurred after the last group session were considered. Participants who missed more than 4 group sessions were excluded to avoid potentially insufficient treatment dose. All outcome variables were compared at the points in time when the respective outcomes were assessed using the linear mixed model assuming a random effect for subjects and fixed effects for group, time and their interaction. The random part was specified by random intercepts and slopes for time to adjust for repeated measures (Bates et al. 2015). Kaplan–Meier curves were plotted to compare the rate of recurrences and to estimate the time from baseline to recurrence. Although RCTs help to minimize confounding (e.g., by applying inclusion and exclusion criteria to study a homogeneous sample), confounders might still lead to false negative or positive results. We therefore controlled for potentially confounding variables (sex, age, bipolar subtype, age at disease onset; number of previous episodes, hospitalizations, psychotropic drugs or missed group sessions; history of rapid cycling, suicide attempts, group or individual psychotherapy; outcome assessments at baseline) by using a multivariate confounder analysis (Harrell 2001).

## Results

### Participants

Patients were randomized and received either the experimental (*n* = 39) or control intervention (*n* = 36). Two participants of the control condition were excluded because they missed more than 4 group sessions. The number of missed group sessions in the experimental intervention (mean ± SD: 0.69 ± 0.92) did not significantly differ (*U* = 749.5, *z* = 1.044, *p* = 0.297) from the control intervention (mean ± SD: 0.94 ± 1.07). Adding the 2 excluded participants showed a similar result (*U* = 827.5, *z* = 1.442, *p* = 0.149). Demographic and clinical characteristics were comparable between groups, but significantly more participants of the control condition had received individual psychotherapy (see Table 1). Twenty (51.3%) participants of the experimental and 20 (58.8%) participants of the control intervention dropped out over the follow-up period of 2 years (*Χ*^*2*^ = 0.417, *df* = 1, *p* = 0.518). The dropout rate remained equally distributed across groups after adding the 2 excluded participants of the control condition (*Χ*^*2*^ = 0.734, *df* = 1, *p* = 0.392). Reasons for dropout were: (a) stopping self-reporting (experimental = 7, control condition = 4), (b) starting psychotherapy (experimental condition = 3), (c) schedule difficulties (control condition = 1), (d) no reason given (experimental = 2, control condition = 4), (e) unknown (i.e., lost contact; experimental = 8, control condition = 11).

**Table 1:** Participant characteristics at baseline

	Experimental intervention	Control intervention	Statistics
Sample size	39	34	
Study site			*Χ*^*2*^ = 0.170, *df* = 2, *p* = 0.919
Berlin	20 (51.3)	19 (55.9)	
Chemnitz	8 (20.5)	6 (17.6)	
Dresden	11 (28.2)	9 (26.5)	
Demographics			
Sex			*Χ*^*2*^ = 0.088, *df* = 1, *p* = 0.766
Females	17 (43.6)	16 (47.1)	
Males	22 (56.4)	18 (52.9)	
Age [years]	44.32 ± 11.63	42.69 ± 12.34	*t* = 0.581, *df* = 71, *p* = 0.563
Clinical data			
Bipolar subtype			*Χ*^*2*^ = 3.840, *df* = 1, *p* = 0.050
Type I	24 (61.5)	28 (82.4)	
Type II	15 (38.5)	6 (17.6)	
Age at disease onset [years]	28.87 ± 11.72	26.68 ± 9.87	*U* = 383.0, *z* = − 0.775, *p* = 0.438
Previous episodes	14.83 ± 23.35	15.47 ± 17.45	*U* = 566.5, *z* = 0.342, *p* = 0.732
Hospitalizations	3.18 ± 4.59	3.21 ± 2.77	*U* = 823.0, *z* = 1.792, *p* = 0.073
History of rapid cycling	4 (10.3)	8 (23.5)	*Χ*^*2*^ = 2.330, *df* = 1, *p* = 0.127
History of suicide attempts	16 (41.0)	11 (32.4)	*Χ*^*2*^ = 0.718, *df* = 1, *p* = 0.397
Psychotropic drugs	1.82 ± 0.89	2.09 ± 1.08	*U* = 746.5, *z* = 0.975, *p* = 0.329
Individual psychotherapy ever received	25 (64.1)	29 (85.3)	*Χ*^*2*^ = 4.237, *df* = 1, *p* = 0.040*
Group psychotherapy ever received	17 (43.6)	12 (35.3)	*Χ*^*2*^ = 0.522, *df* = 1, *p* = 0.470

Data are number (%) or mean ± standard deviation

* *p* < 0.05

### Outcomes

**Fig. 1:**
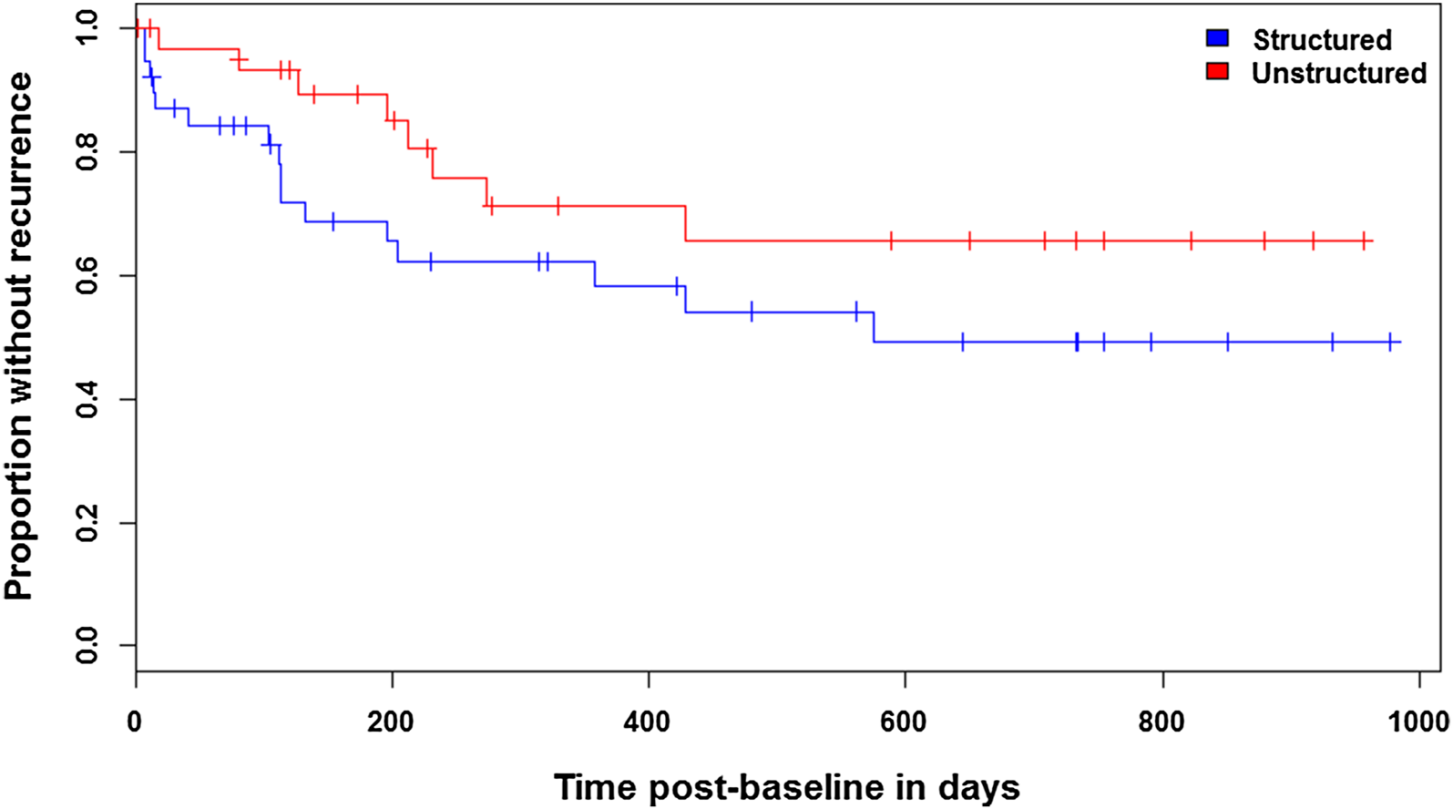
Kaplan-Meier estimates for first recurrence of any type do not differ between groups (*Χ*^*2*^ = 2.215, *df* = 1, *p* = 0.137)

**Fig. 2:**
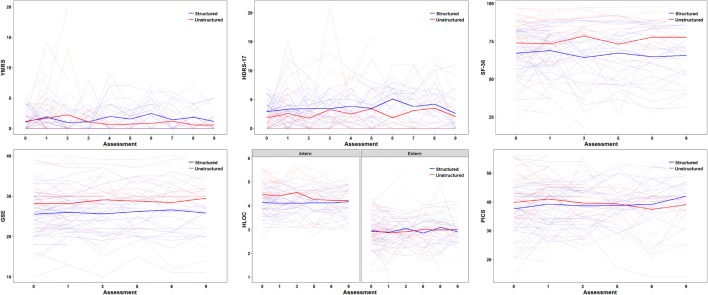
Mood symptoms (YMRS, HRDS-17), quality of life (SF-36), self-efficacy expectations (GSE, HLOC) and perceived involvement in care (PICS) showed no significant group-by-time interactions (smallest *p* = 0.105) and were stable over time independent of group allocation (smallest *p* = 0.105)

After 2 years, 23 patients (59%) in our experimental (*n* = 39) and 15 patients (44%) in our control group (*n* = 34) fulfilled the criteria for recurrence (*Χ*^*2*^ = 1.606, *df* = 1, *p* = 0.205). 41 mood episodes occurred in the experimental group (mean ± SD: 1.05 ± 1.32) compared to 27 in the control group (mean ± SD: 0.79 ± 1.39), without reaching statistical significance (*U* = 550.0, *z* = − 1.356, *p* = 0.175; see Table 2). Time to first recurrence with any mood episode did not significantly differ (*Χ*^*2*^ = 2.215, *df* = 1, *p* = 0.137) between the experimental (25% relapsed after 112 days) and the control group (25% relapsed after 273 days, see Fig. 1). There were no significant group-by-time interactions in mood symptoms as assessed with YMRS (*t* = − 1.185, *df* = 64.1, *p* = 0.241) and HRDS-17 (*t* = 0.558, *df* = 48.4, *p* = 0.580), quality of life as assessed with SF-36 (*t* = 0.922, *df* = 101.7, *p* = 0.359), perceived involvement in care as assessed with PICS (*t* = − 1.171, *df* = 41.1, *p* = 0.248) and self-efficacy expectations as assessed with GSE (*t* = 0.008, *df* = 36.3, *p* = 0.994) and HLOC (*t* = − 1.627, *df* = 460.0, *p* = 0.105). The 3-way interaction between health locus (internal or external), time and group was also not significant (i.e., neither of the loci significantly changed over time between groups; *t* = 1.157, *df* = 509.6, *p* = 0.248). All of these outcomes stayed stable over time independent of group allocation (smallest *p* = 0.105, see Fig. 2). Controlling for potentially confounding variables (sex, age, bipolar subtype, age at disease onset; number of previous episodes, hospitalizations, psychotropic drugs or missed group sessions; history of rapid cycling, suicide attempts, group or individual psychotherapy; outcome assessments at baseline) did not change these results.

**Table 2:** Affective episodes over 2-year follow-up

Bipolar subtype^a^	Experimental intervention		Control intervention	
	I	II	I	II
Sample size	24 (61.5)	15 (38.5)	28 (82.4)	6 (17.6)
At least one recurrence	14	9	12	3
Number of episodes	23	18	15	12
Manic	10	6	8	6
Hypomanic	0	1	0	0
Depressive	5	11	5	6
Mixed	1	0	0	0
Unspecified	7	0	2	0

Data are number (%). ^a^at baseline

## Discussion

The goal of this multicenter, rater-blind RCT was to assess the efficacy of a short group-based PE program (Erfurth et al. 2005) plus subsequent computer-based self-charting (Bauer et al. 2004) for patients with remitted BD. We hypothesized that this combined intervention would result in improved recurrence prevention (i.e., recurrence frequency, time to recurrence), mood stability, quality of life, self-efficacy expectations and perceived involvement in care. However, our study showed no superiority of this structured intervention program plus TAU when compared to an unstructured control condition (matched for duration of group sessions and self-monitoring) plus TAU in any outcomes measured.

Meta-analyses and reviews suggest that PE has gained substantial evidence for efficacy in the long-term treatment of BD over the last years (Bond and Anderson 2015; Macheiner et al. 2017; Miziou et al. 2015; Salcedo et al. 2016; Soo et al. 2018). PE has been delivered in individual settings but it is more frequently delivered in group settings (Bond and Anderson 2015; Salcedo et al. 2016; Soo et al. 2018). Recently, computer and mobile-based PE programs have been developed and studied (Salcedo et al. 2016; Soo et al. 2018). Studies of these different formats have mainly been shown effective as an add-on to TAU (i.e., standard psychiatric care or standard pharmacotherapy for BD; Salcedo et al. 2016). However, RCTs with control interventions that adequately control for non-specific effects of treatment (e.g., by unstructured group meetings) have been scarce (Bond and Anderson 2015). To date, only long-term group PE (21 weeks, 21 × 90 min) plus TAU has demonstrated longer time to recurrence and fewer recurrences in patients with stable remission than an unstructured control condition of similar length plus TAU (Colom et al. 2003a, b, 2009b).

As long-term programs are costly and difficult to implement in clinical practice, we aimed to show that a 6-week PE program (6 × 90 min) can also improve outcomes. However, neither our RCT nor an RCT of an 8-week PE program (16 × 90 min, control sessions employed relaxation techniques; de Barros Pellegrinelli et al. 2013) were able to prove the efficacy of short-term PE in recurrence prevention, reducing mood symptoms or improving quality of life. As both programs basically covered the same topics that were delivered in the long-term PE studies by Colom and Vieta (2006), other factors such as differences in PE length, sample size [N = 120 in Colom et al. (2003a); however, N = 50 in Colom et al. (2003b)] or sample characteristics may explain the discrepant results. After 2 years, 23 patients (59%) in our experimental and 15 patients (44%) in our control group fulfilled the criteria for recurrence; and only 1 subject was hospitalized (*N* = 55) until the end of the 1-year follow-up in the study by de Barros Pellegrinelli et al. (2013). By contrast, recurrence rates of 92% (control) and 60–67% (PE) were recorded at the end of the 2-year follow-up in the studies by Colom et al. (2003a, b). We assessed the criteria for recurrence after the last group session and then every 3 months (counting from first group session). This intermittent examination carries a risk that brief episodes may have been missed. However, we used SCID-I to capture any episodes that might have occurred. Moreover, a review of the literature indicates that the generalizability of the studies by Colom et al. might be limited due to considerably higher recurrence rates compared to other PE studies (Bond and Anderson 2015).

A 5-year follow-up post hoc analysis by Colom et al. (2010) revealed that only patients with up to 6 previous mood episodes showed longer time to recurrence after receiving PE. This finding was echoed by a large multicenter RCT that compared group PE based on the manual by Colom and Vieta (2006) to optimized unstructured peer support (Morriss et al. 2016). PE delayed time to next bipolar episode only in patients with up to 7 previous episodes (Morriss et al. 2016); however, PE was superior to peer support in the overall sample on time to next manic episode, acceptability (defined by number of attended sessions) and interpersonal function as assessed with the interpersonal domain of the Social Adjustment Scale (Morriss et al. 2016). Along those lines, a short group PE (2 weeks, 8 × 40–60 min) showed improved recurrence prevention and clinical global functioning at 1 year in inpatients with remitted mania and few previous bipolar episodes (Chen et al. 2018). Of note, limitations of this study are a sample of 90% females and the delivery of unstructured group meetings by nurses (PE by psychiatrists or clinical psychologists). Taken together, a growing body of evidence suggests that PE may be more efficacious at earlier stages of the disease. The fact that 68% of patients had 6 or more previous episodes in our study (missing information for 10%) and 80% in the study by de Barros Pellegrinelli et al. (2013) may have prevented efficacy of these short-term PE programs. Due to few patients in the early course of their disease, few recurrences and a high dropout rate in our study, we were not able to address this issue by using a post hoc analysis. A multivariate confounder analysis did however not reveal a significant influence of the number of previous episodes on any outcomes measured. Number of previous episodes also did not influence outcomes in a study comparing 6 sessions of group PE (90 min each) to individual cognitive behavioral therapy (20 × 50 min) in which no differences in mood symptoms or recurrence prevention between groups were found (Parikh et al. 2012). Although mood symptoms decreased after both interventions over time, it remains unclear if both conditions were equally effective or ineffective due to a lack of a TAU only condition (Parikh et al. 2012).

The innovative aspect of this trial was the delivery of a short PE program followed by self-monitoring via ChronoRecord, which has not been evaluated to date. Structured self-monitoring was assumed to be more effective than unstructured diary writing in helping patients using the knowledge and coping skills learned in PE in everyday life. As self-reporting was part of the interventions, we did not aim to assess the efficacy of computer-based self-charting itself. Although the benefit of guided self-monitoring is often assumed (e.g., by promoting early intervention), there is actually no good evidence for this assumption (Faurholt-Jepsen et al. 2016). It is even conceivable that rigorous self-monitoring bears harmful effects (e.g., by promoting depressive ruminations; Faurholt-Jepsen et al. 2016) and might thereby even have undermined possible effects of our PE program.

### Limitations

Our study may be seen as limited by pooling patients with bipolar I and II disorders, a high dropout rate and the lack of an additional TAU only condition. The first 2 points can however also be regarded as strengths since they contributed to a real-world evaluation of our program. Moreover, a post hoc analysis of the RCT by Colom et al. (2009a) and an observational study using registry data (Joas et al. 2019) found that PE was also effective in patients with bipolar II disorder. Because patients with bipolar II disorder were numerically overrepresented in our experimental compared to our control group and suffered from more episodes over the 2-year follow-up than those with bipolar I disorder, we included bipolar subtype in a multivariate confounder analysis, which did not change our results. Of note, 8 patients with bipolar II disorder at baseline were diagnosed with mania during the follow-up, but further post hoc analyses were not attempted due to small sample sizes. As 84% of our study patients had already received any kind of psychotherapy at some point, our short PE program may not have been able to provide additional benefit. Moreover, simple PE has been recommended as the minimal aim of any treatment for patients with BD in Germany (Pfennig et al. 2013). As psychoeducational elements should therefore be routinely delivered by psychiatrists, nurses and social workers as part of good clinical practice, there might be a ceiling effect. It is furthermore conceivable that the group setting itself has been the main therapeutic agent in our study. Possible benefits might have been becoming part of a supportive group, sharing information and experience as well as building personal relationships. Lacking therapist lectures, unstructured settings might even offer more space for these factors to take effect. The qualitative study of participant experience by Morriss et al. (2016) underpins these assumptions. Participants reported interacting with other patients suffering from BD as an important reason for study attendance and further participation in the trial (Morriss et al. 2016). Yet the absent improvement of outcome values over time might imply that both interventions were equally ineffective, which cannot be ruled out due to a lack of a TAU only condition in our study. Of note, a TAU only condition may also be subject to pitfalls as a network meta-analysis suggested waiting list being a nocebo (Furukawa et al. 2014). The high dropout rate (55%) over the follow-up period raises the possibility of type II error (i.e., not detecting a genuine superiority of the experimental condition). However, the participants of the control condition relapsed later and less frequently than those of the experimental intervention (although far from reaching statistically significance).

## Conclusions

Six weekly psychoeducational group sessions followed by daily self-monitoring via ChronoRecord for 54 weeks may not be superior to non-structured group meetings followed by unstructured self-reporting. Being part of a supportive group and monitoring one’s disease over the long-term might be beneficial regardless of the conceptualization. As our study sample included a large proportion of patients at later disease stages, it emphasizes that we still know little about what psychological intervention works for them. Further research is warranted to tailor interventions to these patients by identifying (1) the ingredients to which they may respond (e.g., resource-oriented instead of educational focus), (2) how they are best delivered to them (e.g., inpatient or outpatient setting) and (3) whether self-monitoring can play a beneficial part in these interventions.

## Data Availability

The data will not be shared or made publicly available as informed consent for this was not sought from the participants prior to data collection.

## Abbreviations

BD: bipolar disorders
DSM: Diagnostic and Statistical Manual of Mental Disorders
GSE: General Self-Efficacy Scale
HDRS-17: Hamilton Depression Rating Scale
HLOC: Health Locus of Control Scale
PE: psychoeducation
PICS: Perceived Involvement in Care Scales
RCT: randomized controlled trial
SCID: Structured Clinical Interview for DSM-IV
SF-36: 36-Item Short Form Survey
TAU: treatment-as-usual
YMRS: Young Mania Rating Scale
